# Peptide Mimicking Loop II of the Human Epithelial Protein SLURP-2 Enhances the Viability and Migration of Skin Keratinocytes

**DOI:** 10.32607/actanaturae.27494

**Published:** 2024

**Authors:** O. V. Shlepova, T. Ya. Gornostaeva, I. D. Kukushkin, V. N. Azev, M. L. Bychkov, Z. O. Shenkarev, M. P. Kirpichnikov, E. N. Lyukmanova

**Affiliations:** Shemyakin–Ovchinnikov Institute of Bioorganic Chemistry, Moscow, 117997 Russian Federation; Moscow Center for Advanced Studies, Moscow, 123592 Russian Federation; Branch of the Shemyakin–Ovchinnikov Institute of Bioorganic Chemistry, Pushchino, 142290 Russian Federation; Shenzhen MSU–BIT University, Longgang District, Shenzhen, Guangdong Province, 518172 China; Interdisciplinary Scientific and Educational School of Moscow University “Molecular Technologies of the Living Systems and Synthetic Biology”, Department of Biology, Lomonosov Moscow State University, Moscow, 119234 Russian Federation

**Keywords:** SLURP-1, SLURP-2, Ly6/uPAR, nicotinic acetylcholine receptor, keratinocytes, migration, wound healing

## Abstract

The secreted human protein SLURP-2 is a regulator of epithelial homeostasis, which enhances the viability and migration of keratinocytes. The targets of SLURP-2 in keratinocytes are nicotinic and muscarinic acetylcholine receptors. This work is devoted to the search for the SLURP-2 functional regions responsible for enhancing keratinocyte viability and migration. We produced synthetic peptides corresponding to the SLURP-2 loop regions and studied their effect on the viability and migration of HaCaT skin keratinocytes using the WST-8 test and scratch-test, respectively. The highest activity was exhibited by a loop II-mimicking peptide that enhanced the viability of keratinocytes and stimulated their migration. The peptide activity was mediated by interactions with α7- and α3β2-nAChRs and suppression of the p38 MAPK intracellular signaling pathway. Thus, we obtained new data that explain the mechanisms underlying SLURP-2 regulatory activity and indicate the promise of further research into loop II-mimicking peptides as prototypes of wound healing drugs.

## INTRODUCTION

Ly6/uPAR family proteins are expressed in many human tissues and cells [1]. Ly6/uPAR proteins exhibit a wide range of functions and are involved in regulation of cell proliferation, migration, intercellular interactions, immune cell maturation, macrophage activation, and cytokine production. They are also involved in cognitive processes [1, 2, 3]. Some of these proteins are ligands of nicotinic and muscarinic acetylcholine receptors (nAChRs and mAChRs, respectively). Acetylcholine receptors regulate various processes, in particular epithelial cell growth, migration, and differentiation [4, 5]. Acetylcholine receptor ligands may be used as prototypes of drugs effective in diseases arising from dysfunction of these receptors [6, 7]. Human secreted Ly6/uPAR proteins, SLURP-1 and SLURP-2, are auto/paracrine regulators of epithelial homeostasis and ligands of acetylcholine receptors [8, 9, 10, 11]. SLURP-1 inhibits the growth and migration of normal and tumor cells [12, 13, 14, 15]. This protein used to be considered as a prototype of anticancer drugs that target α7-nAChR [12, 16]. The SLURP-2 protein stimulates the proliferation and migration of oral keratinocytes Het-1A and may serve as a prototype of wound-healing drugs [17, 18]. SLURP-2 can interact with the α3, α4, α5, α7, β2, and β4 subunits of nAChR, as well as with M1 and M3 mAChRs. SLURP-2 inhibits current through the ion channel of α4β2- and α3β2-nAChRs, whereas at low concentrations it potentiates α7-nAChR [17]. In this case, SLURP-2 accelerates the migration of Het-1A keratinocytes via interaction with α7-nAChR [18] and stimulates keratinocyte proliferation through interactions with α3β2-nAChR and mAChRs [17]. The replacement of the amino acid residue R20 by alanine at the “head” of the SLURP-2 molecule enhances the inhibition of the current through α7-nAChR and accelerates the migration of keratinocytes [18].

**Fig. 1:**
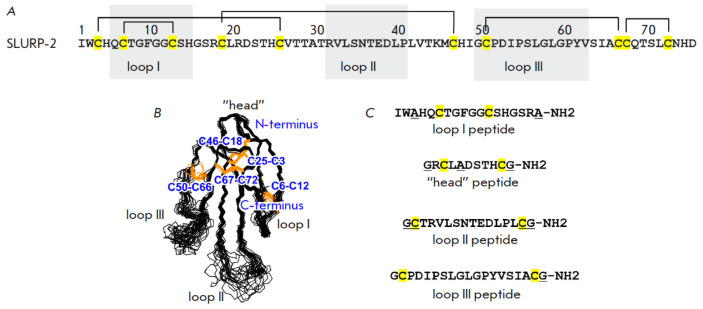
The structures of SLURP-2 and peptides corresponding to the protein loops and “head.” (*A*) Amino acid sequence of SLURP-2. (*B*) Spatial structure of SLURP-2 [17]. (*C*) Amino acid sequence of peptides corresponding to the SLURP-2 loops and “head.” Cysteine residues are shown in yellow or orange, and disulfide bonds are shown as lines connecting two cysteine residues

The functional epitopes of Ly6/uPAR proteins (also called three-finger proteins due to their characteristic three-finger fold, *Fig. 1 A,B*) are loop regions [19]. In this study, we generated synthetic fragments corresponding to the SLURP-2 loop regions and studied how they affect the migration and viability of HaCaT skin keratinocytes. A loop II-mimicking peptide was found to increase keratinocyte viability via interaction with α7-nAChR and stimulate migration via interaction with α3β2-nAChR and inhibition of p38 MAPK activation. The findings of this study suggest that the loop II-mimicking peptide may be a promising wound-healing agent.

## EXPERIMENTAL

**Cell culture**

Human HaCaT cells (immortalized human skin keratinocyte line) were received from the American Type Culture Collection (ATCC, USA). The cells were cultured at 37°C and 5% CO_2_ in a DMEM medium (PanEco, Russia) containing 2 mM L-glutamine and 25 mM glucose and supplemented with 10% fetal bovine serum (Biosera, France), which is designated below as a complete medium. The cells were cultured at 37°C and 5% CO_2_ and were passaged at least twice a week.

**Production of SLURP-2 and its peptide mimetics**

The recombinant SLURP-2 protein was produced in *E. coli *cells as described previously [20]. Peptides mimicking the SLURP-2 first, second, and third loops and head (*Fig. 1B*) were prepared by chemical synthesis according to [15]. The purity and correct spatial structure of (poly)peptides were confirmed by mass spectrometry, high-performance liquid chromatography, and 1H-NMR spectroscopy.

**Effects of SLURP-2, its peptide mimetics, and acetylcholine receptor inhibitors on the viability of HaCaT cells**

The cells were seeded in 96-well plates (5 × 10^3^ cells per well). After 24 h, SLURP-2 or its peptide mimetics at a concentration of 100 nM prepared from a 1 mM stock solution in 100% DMSO by dilution with the complete medium were added to the cells. Then, the cells were incubated at 37°C and 5% CO_2_ for 24 h. The lack of any effect of 0.01% DMSO on cell viability and migration was confirmed in a separate experiment.

To investigate the influence of acetylcholine receptor inhibitors on the effects of SLURP-2 and loop II, HaCaT cells were pre-incubated with atropine (Atr (Sigma-Aldrich, USA), a non-selective mAChR inhibitor), α-conotoxin MII (α-CTxMII (Tocris, UK), a selective α3β2-nAChR inhibitor), dihydro-β-erythroidine (Dhβe (Sigma-Aldrich), a selective α4β2-nAChR inhibitor), and methyllycaconitine (MLA (Sigma-Aldrich), a selective α7-nAChR inhibitor), which were diluted in the complete medium, for 30 min. For all inhibitors, a concentration of 1 μM was used, as determined previously [17]. Next, SLURP-2 or loop II at a concentration of 100 nM and the corresponding inhibitors at a concentration of 1 μM were added to the cells. The cells were then additionally incubated for 24 h.

To assess viability, 5 μL of the CCK-8 reagent (Servicebio, China) were added to the cells, and they were incubated at 37°C and 5% CO_2_ for 1 h. Further, the optical density at 450 nm was measured on a Bio- Rad 680 plate reader (Bio-Rad, USA) and the background value, measured at 655 nm, was subtracted. The resulting data were analyzed using the Graphpad Prism 9.5.0 software (GraphPad Software, USA).

**Effects of SLURP-2, its peptide mimetics, and acetylcholine receptor inhibitors on HaCaT cell migration**

The effects of SLURP-2, its peptide mimetics, and acetylcholine receptor inhibitors (Atr, α-CTxMII, Dhβe, and MLA) on HaCaT cell migration in an *in vitro* wound-healing model (scratch assay) were analyzed using the previously described procedure [15]. HaCaT cells were seeded in 96-well plates (3 × 10^4^ cells/well) and grown at 37°C and 5% CO_2_ for 24 h. Then, a vertical scratch was made with a sterile 10 μL pipette tip (GenFollower tip, E-FTB10S, China). The cells were washed with PBS, and SLURP-2 or its peptide mimetics at a concentration of 100 nM, or receptor inhibitors at a concentration of 1 μM (Atr, α-CTxMII, Dhβe, MLA), alone or mixed with SLURP-2 or the loop II-mimicking peptide, diluted from a 1 mM stock solution in 100% DMSO using a serum-free medium, were added to the cells. Images of the wells with scratched cell monolayers were analyzed after 0 and 24 h at 20× magnification using a CloneSelect Imager cell analysis system (Molecular Devices, USA). The images were digitized, and the scratch area was estimated by calculating the percentage of scratch area covered by migrating cells using the ImageJ (NIH, USA) and MS Excel (Microsoft, USA) software. The results were analyzed using the Graphpad Prism 9.5.0 software (GraphPad Software).

**Real-time PCR**

Total mRNA was extracted from the cultured cells using a HiPure Total RNA Plus kit (Magen, China) according to the manufacturer’s instructions. Total cDNA was synthesized using a MINT Reverse Transcriptase kit (Evrogen, Russia) according to the manufacturer’s protocol. Next, real-time PCR was performed using the primers listed in *Table 1* and a ready-to-use mixture for quantitative PCR that contained a fluorescent dye SYBR Green I from a 5X qPCRmix-HS SYBR kit (Evrogen).

The negative controls contained all PCR mixture components, except cDNA, and did not produce a signal. All PCR reactions were performed using a Roche Light cycler 96 with real-time detection. Data were analyzed using the Light-Cycler 96 SW1.01 software. Gene expression levels were normalized to the expression levels of the housekeeping gene *RPL13A*.

**Protein phosphorylation analysis**

**Table 1:** The oligonucleotide primers used in the study

Gene	Oligonucleotide sequence	
	Forward primer	Reverse primer
RPL13A	TCAAAGCCTTCGCTAGTCTCC	GGCTCTTTTTGCCCGTATGC
ITGA1	ATAAGTGGCCCAGCCAGAGA	CAGCAGCGTAGAACAACAGTG
ITGA2	CGGTTATTCAGGCTCACCGA	GCTGACCCAAAATGCCCTCT
ITGA3	CCTGCACCCCAAAAACATCA	AGGTCCTGCCACCCATCATT
ITGA5	GGGCTTCAACTTAGACGCGGA	CCCCAAGGACAGAGGTAGACA
ITGA6	GGTGGAGAGACTGAGCATGA	GTCAAAAACAGCAGGCCTAAGTA
ITGA9	GACCGCGATGATGAGTGGAT	GATGAGCACAGGCCAACACA
ITGAV	GACTCCTGCTACCTCTGTGC	GAAGAAACATCCGGGAAGACG
ITGB1	CCGCGCGGAAAAGATGAAT	CCACAATTTGGCCCTGCTTG
ITGB3	ATTGGAGACACGGTGAGCTT	ACTCAAAGGTCCCATTGCCA
SNAI1	GGTTCTTCTGCGCTACTGCT	TGCTGGAAGGTAAACTCTGGAT
SNAI2	ACTGGACACACATACAGTGATT	ACTCACTCGCCCCAAAGATG

Phosphorylation of cellular signaling proteins was analyzed using Bio-Plex magnetic particles (Bio-Rad, USA). Cells were incubated with 100 nM SLURP-2 or loop II prepared from a 1 mM stock solution in 100% DMSO by dilution with the complete medium, for 24 h. Then, the cells were lysed using buffer provided by the manufacturer. Analysis was performed on a Bio-Rad 200 flow cytometer (Bio-Rad) according to the manufacturer’s instructions and using the Bio- Plex Manager 6.2 software (Bio-Rad).

**Statistical data processing**

**Fig. 2:**
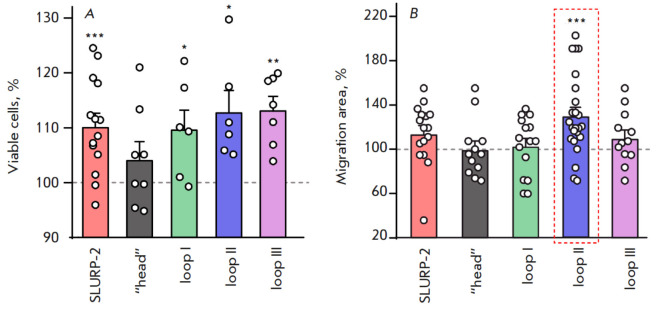
The effects of SLURP-2 and peptides on the viability and migration of HaCaT keratinocytes. (*A*) Effects of 100 nM SLURP-2 and peptides on the viability of HaCaT keratinocytes. Data are shown as percentage of control ± standard error of the mean (*n *= 6–14); 100% of viable cells corresponds to untreated cells. * *p* < 0.05, ** *p* < 0.01, and *** *p* < 0.001 indicate a significant difference from the control (100%) according to the one-sample Student’s *t*-test. (*B*) Effects of 100 nM SLURP-2 and peptides on the migration of HaCaT keratinocytes. Data are shown as a percentage of the control ± standard error of the mean (*n *= 12–24); 100% corresponds to untreated cells. *** *p* < 0.001 indicates a significant difference from the control (100%) according to the one-sample Student’s *t*-test

**Fig. 3:**
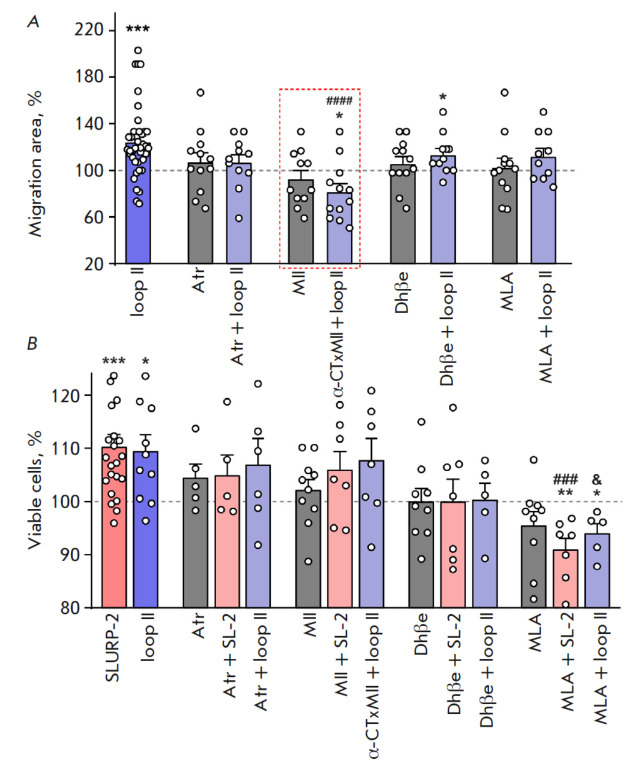
The effects of SLURP-2, peptides, and inhibitors of different acetylcholine receptors on the viability and migration of HaCaT keratinocytes. (*A*) Effects of the loop II peptide (100 nm) and inhibitors of different acetylcholine receptors (1 μM) on the migration of HaCaT keratinocytes. Data are shown as a percentage of the control ± standard error of the mean (*n *= 11–40); 100% corresponds to the migration area of untreated cells. * *p* < 0.05 and *** *p* < 0.001 indicate a significant difference from the control (100%) according to the one-sample Student’s *t*-test. #### *p* < 0.001 indicates a difference from the loop II group according to the one-way ANOVA test, followed by the Dunnett’s/ hoc test. (*B*) Effects of inhibitors of different acetylcholine receptors on the activity of SLURP-2 and the loop II peptide. Data are shown as a percentage of the control ± standard error of the mean (*n *= 4–14); 100% of viable cells corresponds to untreated cells. * *p* < 0.05, ** *p* < 0.01, and *** *p* < 0.001 indicate a significant difference from the control (100%) according to the one-sample Student’s* t*-test. ### *p* < 0.001 indicates a difference from the SLURP-2 group according to the one-way ANOVA test, followed by the Dunnett’s/hoc test; & *p* < 0.05 indicates a difference from the loop II group according to the one-way ANOVA test followed by the Dunnett’s/hoc test

**Fig. 4:**
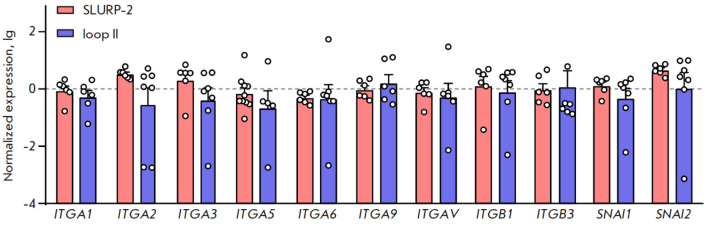
The effects of 100 nM SLURP-2 and loop II peptide on the expression of mRNAs encoding α1, α2, α3, α5, α6, α9, β1, and β3 integrins and the SNAI1 and SNAI2 transcription factors. Data are normalized to the mean expression value in untreated cells and shown as lg ± standard error of the mean (*n *= 5–10). Gene expression is normalized to that of the housekeeping gene *RPL13A*

**Fig. 5:**
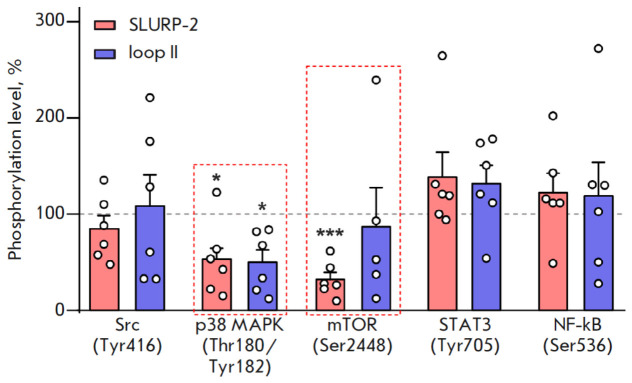
The effects of 100 nM SLURP-2 and loop II peptide on phosphorylation of signaling proteins: Src (Tyr416), p38 MAPK (Thr180/Tyr182), mTOR (Ser2448), STAT3 (Tyr705), and NF-kB (Ser536). Data are shown as a percentage of the control ± standard error of the mean (*n *= 5 to 6); 100% corresponds to the phosphorylation level in untreated cells. * *p* < 0.05 and *** *p* < 0.001 indicate a significant difference from the control (100%) according to the one-sample Student’s* t*-test

Data are presented as a mean ± standard error of the mean (SEM). The number of samples (*n*) is shown in the figure legends. Statistical analysis was performed using the GraphPad Prism 9.5.0 software. Normality of the distribution was assessed using the Shapiro– Wilk test. The analysis was performed using the one-sample Student’s *t*-test (in the case of comparison with the normalized control, *Figs. 2 - 5*) and the oneway ANOVA test, followed by the Dunnett’s test (in the case of multiple comparisons, *Fig. 3*). Differences between groups were considered statistically significant at *p* < 0.05.

## RESULTS AND DISCUSSION

**SLURP-2 loops I, II, and III are important for enhancing skin keratinocyte viability**

The loops of Ly6/uPAR proteins are considered functional epitopes responsible for the activity of three-finger proteins [19]. Previously, we showed that a SLURP-1 loop I-mimicking peptide exhibited similar antitumor activity as the full-length protein [15, 16]. In the present study, we decided to identify the SLURP-2 regions responsible for its activity, namely, for enhancing the viability and stimulating the migration of keratinocytes, which had been shown previously [17, 18, 21]. For this purpose, peptides containing SLURP-2 loop I-, II-, and III-mimicking regions as well as the “head” of the molecule (*Fig. 1*) were prepared using chemical synthesis.

Investigation of the effects of SLURP-2 and the peptides on the viability of HaCaT skin keratinocytes revealed that SLURP-2 increased keratinocyte viability (*Fig. 2A*). In this case, the “head” peptide did not affect keratinocyte viability, whereas loops I-, II-, and III-mimicking peptides stimulated the viability of keratinocytes, similarly to the effect of the full-length SLURP-2 (*Fig. 2A*).

Thus, loops I, II, and III are important SLURP-2 regions required for enhancing the viability and, possibly, proliferation of keratinocytes. The lack of any activity of the SLURP-2 “head” peptide indicates that this region of the full-length protein is not involved in the interaction with the target responsible for stimulating keratinocyte viability. Perhaps, the inactive “head” compensates for the increased activity of loop II whereas the activity of loops I and III is similar to that of the full-length protein. This suggestion is supported by the fact that the replacement of amino acid residue R20 by alanine in the SLURP-2 “head” leads to the stimulation of keratinocyte migration [18].

The SLURP-1 protein had been anticipated to interact simultaneously with different targets: α7-nAChR and the epidermal growth factor receptor [15]. In this case, the interaction with the second target was mediated by the SLURP-1 “head.” Probably, the situation is similar in the case of SLURP-2, where loop II and the “head” interact with different targets, compensating for their influence on the viability of keratinocytes. It is noteworthy that, unlike SLURP-2, the epithelial protein SLURP-1 does not increase but decreases the viability of oral keratinocytes Het-1A, and that its functional region is loop I [22].

**Loop II activates the migration of skin keratinocytes via interaction with α3β2-nAChR**

Previously, SLURP-2 was shown to enhance the migration of Het-1A keratinocytes via interaction with α7-nAChR [18]. In the present work, we studied the effects of SLURP-2 and its peptide mimetics on the migration of HaCaT skin keratinocytes. SLURP-2, loops I and III, and the “head” were found not to exert a significant effect on the migration of HaCaT keratinocytes (*Fig. 2B*) in a scratch closure model. In this case, loop II accelerated keratinocyte migration by ~30% (*Fig. 2B*). Probably, SLURP-2, interacting with different acetylcholine receptor subtypes, is able to both increase and decrease cell migration, with the overall effect dependent on the expression of certain receptors in specific cells.

SLURP-2 is known to interact with the nAChR α3, α4, α5, α7, β2, and β4 subunits and M1 and M3 mAChRs [17]. To elucidate the interaction with which receptor is responsible for the stimulating effect of SLURP-2 loop II on the keratinocyte migration, the effect of loop II was studied in the presence of inhibitors of different acetylcholine receptor subtypes: atropine (Atr), a non-selective mAChR inhibitor; α-conotoxin MII (α-CTxMII), a selective α3β2-nAChR inhibitor; dihydro-β-erythroidine hydrobromide (Dhβe), a selective α2β4-nAChR inhibitor; and methyllycaconitine (MLA), a selective α7-nAChR inhibitor. We demonstrated that inhibition of α3β2-nAChR by α-CTxMII canceled the effect of loop II on HaCaT keratinocyte migration. Concomitant use of atropine and Dhβe with loop II did not significantly affect migration, with the obtained values being not significantly different from the effect of loop II. The obtained data do not indicate whether mAChR and α2β4-nAChR are involved in the effect of loop II on migration (*Fig. 3A*). Thus, loop II stimulates skin keratinocyte migration via the interaction with α3β2-nAChR and, possibly, mAChR and α2β4-nAChR. It is worth noting that in a previously constructed model of the SLURP-2–α3β2-nAChR interaction, loop II was the main SLURP-2 region interacting with this receptor and forming the largest number of contacts in the complex [17]. In this case, inhibitors of other acetylcholine receptors did not significantly affect the effect of loop II.

**The effects of SLURP-2 and loop II on skin keratinocyte viability are mediated by the interaction with α7-nAChR**

In this work, we also studied the influence of inhibitors of different acetylcholine receptor subtypes (atropine, α-conotoxin MII, Dhβe, and MLA) on the effect of SLURP-2 and loop II on keratinocyte viability. Pre-incubation of the cells with MLA was shown to completely abolish the stimulating effect of SLURP-2 and loop II on the viability of HaCaT cells (*Fig. 3B*). However, none of the inhibitors, except MLA, had a significant effect on the activity of SLURP-2 and loop II. In this case, atropine, α-conotoxin MII, and Dhβe, together with SLURP-2 and loop II, did not significantly increase the viability compared to that in the control. Therefore, the contribution of mAChR, α3β2-nAChR, and α2β4-nAChR to the effects of SLURP-2 and loop II on viability requires further research. Thus, the ability of the SLURP-2 protein and loop II peptide to enhance keratinocyte viability is mediated by the interaction with α7-nAChR and, probably, mAChR, α3β2-nAChR, and α2β4-nAChR.

However, SLURP-2 has been previously shown to enhance the viability of Het-1A oral keratinocytes through interaction with α3β2-nAChR, but not with α7-nAChR [17]. Involvement of different receptors in the regulation of SLURP-2 activity in oral and skin keratinocytes may be associated with the different expression profiles of certain receptors in different cells and tissues of the body and lies within the framework of the “polygamous” activity of the epithelial protein that is able to interact with various acetylcholine receptors [17].

**The effects of SLURP-2 and loop II on the viability and migration of skin keratinocytes are not associated with altered expression of integrins and SNAI transcription factors**

Integrins are known to regulate adhesion, migration, and proliferation of epithelial cells, in particular skin keratinocytes [23, 24]. Also, the factors that regulate the migration and differentiation of keratinocytes include the SNAI1 and SNAI2 transcription factors [25]. We ventured that the effects of SLURP-2 and loop II on viability and migration may be related to the influence on expression of integrins or SNAI transcription factors. However, we did not find any significant changes in the expression of the *ITGA1*, *ITGA2*,* ITGΑ3*, *ITGA5*, *ITGA6*, *ITGA9*, *ITGB1*, *ITGB3*, *SNAI1*, and *SNAI2 *genes in HaCaT keratinocytes after incubation with SLURP-2 or loop II for 24 h compared to that in the control (untreated cells, *Fig. 4*). Thus, the effects of SLURP-2 and loop II on the viability and migration of HaCaT keratinocytes are not related to changes in the expression of the genes encoding integrins and SNAI1, or SNAI2 transcription factors.

**The effects of SLURP-2 and loop II on skin keratinocytes are related to the suppression of the p38 MAPK and mTOR signaling pathways**

Previously, the SLURP-1 protein was shown to inhibit the activity of intracellular signaling cascades associated with AKT, PTEN phosphatase, and mTOR protein kinase in tumor cells [15]. We venture that the effects of SLURP-2 and loop II could also be related to the regulation of the intracellular signaling cascades associated with proliferation and migration. In addition, we investigated the effects of SLURP-2 and loop II on the activity of the STAT3 and NF-kB transcription factors involved in the regulation of gene expression in epithelial cells and associated with α7-nAChR activation [26, 27, 28, 29]. Using the Bioplex magnetic bead array analysis, we showed that both SLURP-2 and the loop II peptide inhibited phosphorylation and, therefore, the activation of p38 MAPK kinase in HaCaT keratinocytes after 24-h incubation (*Fig. 5*). Furthermore, SLURP-2 – but not loop II – reduced mTOR kinase phosphorylation in HaCaT keratinocytes (*Fig. 5*).

It is known that p38 MAPK activation can cause keratinocyte apoptosis and, therefore, decrease the number of viable cells [30, 31]. At the same time, α7-nAChR activation inhibits p38 MAPK phosphorylation and activation [32]. Previously, SLURP-2, at a concentration of 100 nM, was shown to potentiate α7-nAChR in the presence of acetylcholine [17]. Thus, we may suggest potentiation of α7-nAChR in HaCaT keratinocytes in the presence of 100 nM SLURP-2, which in turn leads to the suppression of the p38 MAPK signaling pathway and an increase in the number of viable cells. This suggestion is consistent with a previously proposed model of the SLURP-2–α7-nAChR interaction where SLURP-2 loop II interacts with the open (active) state of α7-nAChR [17]. This also indicates that activation of this receptor is associated with suppression of the p38 MAPK signaling pathway, with prevention of the apoptosis of HaCaT keratinocytes and an increase in the number of viable cells in the presence of loop II.

It is worth noting that inhibition of mTOR phosphorylation can lead to suppression of keratinocyte migration [33]. Yet we failed to uncover any significant effects of SLURP-2 on migration (*Fig. 2B*). In this case, loop II stimulates migration via the interaction with α3β2-nAChR (*Fig. 3*) and does not inhibit the intracellular signaling cascade associated with mTOR (*Fig. 5*). Probably, other SLURP-2 regions (not loop II) are involved in the inhibition of mTOR phosphorylation, which contributes negatively to migration stimulation by the full-length protein.

Incubation of oral keratinocytes Het-1A with SLURP-1 was previously shown to activate the transcription factor NF-kB [34]. SLURP-1 is known to be a negative modulator of α7-nAChR [35]. Thus, the lack of a potentiating effect on NF-kB phosphorylation in the presence of both SLURP-2 and loop II supports our suggestion that both of these molecules potentiate α7-nAChR at the tested concentration in HaCaT keratinocytes. This is consistent with suppression of the p38 MAPK signaling pathway (*Fig. 5*).

## CONCLUSION

In this study, we produced synthetic peptides corresponding to the SLURP-2 loop fragments (“head”, loop I, loop II, and loop III peptides) and investigated how they affect the viability and migration of skin keratinocytes. The “head” peptide did not affect either the viability or the migration of keratinocytes. Loop I- and loop III-mimicking peptides were shown to increase the viability and to not affect the migration of keratinocytes. The loop II-mimicking peptide was found to exhibit the highest activity. It stimulated both the viability and migration of keratinocytes through the interaction with α7-nAChR and α3β2-nAChR, respectively. In this case, the SLURP-2 protein itself was shown to increase only the viability of keratinocytes and to not affect their migration. The differences in effects of SLURP-2 and loop II on HaCaT keratinocyte viability and migration are likely linked to the ability of the full-length protein to interact with several targets simultaneously, as well as with inhibition of mTOR phosphorylation, which is not relevant to loop II. Thus, we have gained new knowledge about the regulation of epithelial cell homeostasis by the human epithelial protein SLURP-2. Our findings indicate prospects for further research into the properties of loop II and its potential as a prototype for the development of new wound-healing drugs.

## Abbreviations

α-Bgtx: α-bungarotoxin
ACh: acetylcholine
Atr: atropine
Dhβe: dihydro-β-erythroidine hydrobromide
mAChR: muscarinic acetylcholine receptor
Mec: mecamylamine
MII: α-conotoxin MII
MLA: methyllycaconitine
mTOR: mammalian target of rapamycin
nAChR: nicotinic acetylcholine receptor
Nf-kB: nuclear factor kB
p38 MAPK: mitogen-activated protein kinase p38
Src: non-receptor tyrosine kinase Src
STAT3: signal transducer and activator of transcription 3
